# Sublethal Clothianidin Exposure Increases Trait Variation and Drives Sex‐Specific Selection in the Water Strider *Gerris latiabdominis*

**DOI:** 10.1002/ece3.74223

**Published:** 2026-08-23

**Authors:** Byeongho Lee, Jiman Heo, Chang S. Han

**Affiliations:** ^1^ Department of Biology Kyung Hee University Seoul Korea; ^2^ Korea Institute of Ornithology Kyung Hee University Seoul Korea

**Keywords:** agroecosystem, clothianidin, insecticide, semi‐aquatic insect, sexual dimorphism, sublethal effects

## Abstract

Agricultural insecticides pose significant risks to non‐target insects, extending beyond immediate mortality to sublethal effects that can alter evolutionary trajectories. While sublethal exposure may reduce average trait expression, it can also unmask hidden genetic variation, thereby increasing trait variance. Moreover, if coupled with sex‐specific selection, such exposure could reshape sexual dimorphism. However, the role of sublethal insecticide exposure in driving these evolutionary dynamics remains poorly understood, particularly for semi‐aquatic insects in agroecosystems. Here, we investigated how sublethal exposure to clothianidin, a widely used neonicotinoid, affects trait means, variance, and selection in male and female water striders ( *Gerris latiabdominis* ). We monitored lifespan and repeatedly measured body mass and activity of adults exposed to a sublethal concentration of clothianidin during their final nymphal stage, comparing with unexposed controls. Clothianidin exposure increased adult mortality risk and reduced body mass (male: −8.3%; female: −8.5%). Importantly, exposure substantially increased among‐individual variance in body mass (male: 222%; female: 326%) and lifespan (male: 111%; female: 203%). Although these changes in means and variances were not statistically sex‐specific, the increase in variance was more pronounced in females. In addition, positive viability selection on body mass was observed only in exposed females. These findings suggest that sublethal clothianidin exposure has the potential to influence the evolution of increased sexual dimorphism in body mass. Our results highlight the importance of considering insecticide effects on trait variance and selection—not merely trait means—to fully understand evolutionary changes in non‐target populations. We suggest incorporating sublethal effects on semi‐aquatic predatory insects into ecological risk assessments on agroecosystems.

## Graphical abstract

We highlight the importance of considering insecticide effects on trait variance and selection—not merely trait means—to fully understand evolutionary changes in non‐target populations. We suggest incorporating sublethal effects on semi‐aquatic predatory insects into ecological risk assessments on agroecosystems.

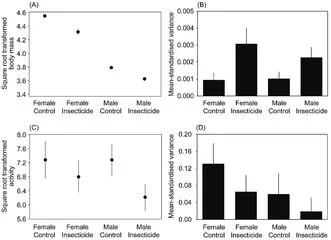

## 1 Introduction

Insecticides are highly effective tools for pest control, regulating populations by directly reducing fecundity and increasing mortality (Guedes et al. 2016). However, they can also harm non‐target species, including natural enemies and pollinators, which perform crucial ecological roles (Desneux et al. 2007; Köhler and Triebskorn 2013; Miles et al. 2017). Moreover, beyond acute lethality, sublethal exposure can profoundly alter the expression of behavioural, physiological, and morphological traits in non‐target insects. These sublethal effects entail broader ecological risks that extend beyond immediate pest control (Desneux et al. 2007; Serrão et al. 2022).

Sublethal exposure to insecticides can affect both the mean and variance of traits within a single generation (e.g., Royauté et al. 2015; Tüzün et al. 2017), potentially influencing selection and driving trait evolution across generations. Environmental stressors, such as insecticides, can reveal hidden genetic variance, leading to increased trait variance (Badyaev 2005; Brand et al. 2023; Paaby and Rockman 2014; Rowiński and Rogell 2017; Schlichting 2008). This increase in variance is important for trait evolution because it provides the raw material upon which selection can act and increases the potential rate of evolutionary change. Furthermore, traits linked to insecticide resistance are likely to be under different levels of selection depending insecticide exposure. For example, higher metabolic rates have been shown to enhance survival in blue‐tailed damselflies (*Ischnura elegans*) exposed to insecticides (Janssens et al. 2021), suggesting that such traits may play critical roles in detoxification. Thus, insecticides can simultaneously shape trait distributions and impose selection on specific traits in non‐target insects, thereby facilitating their rapid evolution.

Moreover, sublethal insecticide exposure may exert sex‐specific effects on both trait variance and selection acting on sexually homologous traits. For example, male jumping spiders ( *Eris militaris* ) exposed to the insecticide phosmet showed increased within‐individual variation in activity but unchanged within‐individual variation in prey capture behaviour (Royauté et al. 2015). In contrast, females showed the opposite pattern: increased variation in prey capture behaviour but unchanged variation in activity (Royauté et al. 2015). In addition, selection on shared traits often differs between sexes (Singh and Punzalan 2018), driving the evolution of sexual dimorphism (Clutton‐Brock 2007; Schärer et al. 2012). As the strength and outcome of sex‐specific selection may depend on environmental stress (reviewed by De Lisle et al. 2018), insecticide‐induced changes in sex‐specific selection could consequently influence the evolution of sex differences in traits over multiple generations.

Despite this possibility, few studies have comprehensively explored how sublethal insecticide exposure simultaneously affects trait means, variances, and selection in a sex‐specific manner. These sex‐specific effects are critical, as they could drive evolutionary divergence between males and females, thereby shaping sexual dimorphism (Lande 1980). Even when sublethal insecticide exposure does not alter trait means differently between sexes within a single generation, its sex‐specific effects on trait variance and selection could still cumulatively affect sexual dimorphism across generations (Bonduriansky and Chenoweth 2009; Cheng and Houle 2020; Cheverud et al. 1985; Connallon and Clark 2014; Fisher 1958; Lande 1980, 1987; Lynch and Walsh 1998; Wyman et al. 2013).

In this study, we examined the effects of sublethal exposure to clothianidin, a widely used neonicotinoid insecticide, on the water strider *Gerris latiabdominis* (Heteroptera: Gerridae). *G. latiabdominis* , a semi‐aquatic insect widely distributed in East Asia, inhabits shallow water bodies such as paddy fields, ponds, and slow‐moving creeks (Nam et al. 2019; Ye et al. 2020), feeding on insects trapped on the water surface (Spence and Andersen 1994). While paddy fields provide stable habitats with abundant water and prey for *G. latiabdominis* , they also pose risks of exposure to insecticides. Particularly, clothianidin is commonly sprayed for pest control during April–May (the period *of G. latiabdominis* adult reproduction) and June–July (the period of *G. latiabdominis* nymphal development) (Ha et al. 2016; Oh et al. 2021). In addition, as neonicotinoids can accumulate across trophic levels—from plants to primary consumers and ultimately to predators (Tooker and Pearsons 2021)—semi‐aquatic predators such as water striders are vulnerable to both direct exposure and indirect ingestion via contaminated prey. Indeed, *Gerris lacustris* populations have been shown to decline in mesocosms treated with clothianidin (Hashimoto et al. 2020). Despite this apparent susceptibility, numerous studies on the sublethal impacts of neonicotinoids have predominantly focused on fully aquatic insects (Bartlett et al. 2018; Maloney et al. 2018; Miles et al. 2017; Morrissey et al. 2015; Sánchez‐Bayo et al. 2016), whereas the insecticide effects on non‐target semi‐aquatic insects living in agroecosystems remain largely unexplored.

Here, we investigated the effects of sublethal clothianidin exposure on trait means, variances, and phenotypic selection in adult *G. latiabdominis* . Specifically, we exposed individuals during their final nymphal stage to mirror the early‐summer (June–July) agricultural application schedule in Korea and assessed whether these effects differ between the sexes. We focused on lifespan and two labile traits: body mass and activity. Body mass, a proxy for energy reserves, is highly responsive to environmental stress (Lease and Wolf 2011; Rowiński and Rogell 2017). Activity, which is often linked to metabolic rate (Videlier et al. 2019, 2021), reflects an individual's physiological state (Videlier et al. 2019, 2021). To accurately assess selection on these labile traits, we partitioned phenotypic variance into among‐individual and within‐individual components, evaluating individual‐level associations between these traits and lifespan (Dingemanse et al. 2021). In insect species with female‐biased sexual size dimorphism, environmental stress typically impacts females more severely than males (Teder and Kaasik 2023). Given that *G. latiabdominis* females are larger than males, we predicted that they would be more vulnerable to sublethal clothianidin exposure. Specifically, the physiological costs associated with coping with insecticide toxicity during development are likely greater for females, as their larger body size necessitates higher resource acquisition (Jones et al. 2009). Consequently, we hypothesised that clothianidin exposure during the final nymphal stage would lead to a more pronounced reduction in trait expression and a greater increase in trait variance in adult females compared to adult males. Furthermore, we predicted that this female‐biased vulnerability would impose stronger survival selection on traits in females.

## 2 Materials and Methods

### 2.1 Rearing Conditions

We collected *G. latiabdominis* using sweep nets from a creek near Sikjang Mountain, Korea, on 21 April 2023. This creek is located in a semi‐natural area with minimal surrounding agricultural activity, indicating a low likelihood of prior insecticide exposure or the development of insecticide resistance in the collected population. The collected individuals were transported to a climate‐controlled room and housed separately in two large plastic tanks (520 × 385 × 180 mm; water depth: 8 cm) equipped with floating pieces of Styrofoam (50 × 20 mm) for resting and oviposition. Water in the tanks was changed weekly. Eggs laid by females on floating Styrofoam were transferred to separate tanks for rearing. The new generation was reared in 6 large plastic tanks (520 × 385 × 180 mm; water depth: 8 cm), with 100 nymphs per tank, and water was changed weekly.

From the 5th instar onwards, nymphs from both the control and insecticide treatments were individually housed in round plastic containers (120 mm diameter × 80 mm height; water depth: 4 cm) and maintained under the same environmental conditions as their parent generation. All individuals, both wild‐caught and laboratory‐reared, were kept at 25°C under a 17L:7D photoperiod and were fed ad libitum with frozen crickets (*Gryllus bimaculatus*).

### 2.2 Insecticide Exposure Protocol

When the nymphs reached 5th instars, their sex was determined based on genitalia morphology, and individuals (87 males and 94 females) were randomly assigned to one of two groups (control/insecticide) for 1 week. Individuals in one group were reared individually in containers (120 mm diameter × 80 mm height) filled with 100 mL of tap water (control group: 44 males and 47 females). Individuals in the other group were similarly housed individually, but each container contained 100 mL of a clothianidin solution (insecticide group: 43 males and 47 females). During the exposure (treatment) period, water was not changed, and individuals were fed ad libitum every 2 days. For the insecticide group, we prepared a stock solution of 10 μg/L clothianidin using a commercially available insecticide (8% clothianidin with surfactant, Hankooksamgong), diluting the concentration to 7 μg/L clothianidin. To ensure uniform exposure and minimise container‐level heterogeneity, a single, thoroughly mixed stock solution was prepared, and exactly 100 mL was subsequently dispensed into each identical container. This insecticide concentration (7 μg/L) is ecologically relevant, as it falls within the estimated exposure concentration of 30 μg/L in paddy fields in Korea (Oh et al. 2021) and is below the LC50 value of 35 μg/L that we identified for water strider nymphs (see Text S1 and Table S1).

During the exposure period, 4 individuals (1 male and 3 females) in the control group and 14 individuals (6 males and 8 females) in the insecticide group died before the behavioural assay was performed, suggesting that the insecticide concentration was mildly harmful to the nymphs (Fisher's exact test, male: *p* = 0.06; female: *p* = 0.20). Additionally, when adult water striders were exposed to 0–25 μg/L solutions of both pure clothianidin and the insecticide formulation used in this study (8% clothianidin with surfactant), the mortality and paralysis rates were similar (see Table S2 and Text S2). Thus, although pure clothianidin and the insecticide formulation may exert somewhat different effects on nymphs, we tentatively interpreted the observed effects of the sublethal insecticide concentration as being primarily driven by clothianidin. However, the potential effect of the surfactant on water striders (e.g., Mullin et al. 2016) warrants caution when interpreting the effects of clothianidin in this study.

After 1 week of exposure, each individual was transferred to an individual container (diameter: 120 mm; height: 80 mm; water depth: 4 cm) filled with tap water for a recovery period. All nymphs moulted into adults during the exposure period. Throughout the treatment and recovery periods, all individuals were maintained under consistent rearing conditions.

### 2.3 Activity, Body Mass and Lifespan Measurements

After a one‐week recovery period, a total of 163 individuals (control group: 43 males and 44 females; insecticide group: 37 males and 39 females) were subjected to a series of assays to measure activity, body mass, and lifespan. Each individual's activity was measured three times, and body mass was estimated four times at 4‐day intervals. For the activity assay, each individual was transferred from its rearing container to an experimental container (230 × 175 × 100 mm; water depth: 40 mm) and allowed a 2‐min acclimation period. This brief acclimation has previously been shown to be sufficient to minimise handling stress and allow individuals to resume normal baseline behaviour (Han and Brooks 2013, 2014, 2015; Han and Jablonski 2019). We then measured the total distance moved during a 10‐min period using EthoVision tracking software (Noldus EthoVision XT 14, Noldus Information Technology). Body mass was estimated to the nearest 0.1 mg after the activity assay using an electronic balance (CATX 124, CAS). Survival was checked every 4 days, and lifespan was calculated from the start of the recovery period until death.

### 2.4 Statistical Analyses

To assess the effects of sublethal clothianidin exposure on lifespan, trait means, among‐individual variance, and selection, we fitted a series of univariate and multivariate mixed‐effects models in Asreml 4.1 (Butler et al. 2009) and *brms* package in R version 4.4 (R Core Team 2024). We also conducted survival analyses using a Cox proportional‐hazards model with *coxme* package in R version 4.4 (R Core Team 2024). We provide the details of the statistical analyses in the Text S3.

To summarise, first, to test insecticide effects on lifespan, lifespan data were analysed using Cox proportional‐hazards models for interval‐censored observations. The model included treatment, sex, and their interaction as independent variables. Next, to test insecticide effects on mean‐level traits (body mass or activity), we used univariate mixed‐effects models. In the model, the focal trait was fitted as the response variable, with testing order, sex, treatment, and the sex‐by‐treatment interaction included as fixed effects. Individual identity was included as a random effect.

To test the effects of sex and insecticide treatment on phenotypic variance in lifespan, we used double‐hierarchical generalised linear models (DHGLMs) fitted to sex‐ and treatment‐specific mean‐standardised data. By including fixed effects for sex, treatment, and their interaction in the model's dispersion component, we were able to directly quantify their impact on phenotypic variance independent of mean differences (see details in Text S3). In the absence of strong prior information, we implemented the default priors provided by the *brms* package (Bürkner 2017). Specifically, group‐level effects (i.e., coefficients for sex, treatment, and their interaction) for both the mean and the residual standard deviation were assigned improper flat priors. The intercepts for both models were assigned weakly informative Student‐t priors.

In addition, to assess the effects of sex and treatment on the extent of among‐individual variance in body mass or activity, we compared a series of multivariate mixed‐effects models (Table 1). In the model, we considered each mean‐standardised trait measured for each unique combination for sex and treatment as a separate response variable in our analyses. The models did not include fixed effects but included individual identity as a random effect. To test whether mean‐standardised among‐individual variances differed between sexes or treatments, we compared a null model in which all the mean‐standardised among‐individual variances were constrained to be equal (model 1 in Table 1) with a model in which mean‐standardised among‐individual variances were constrained to be equal within sexes (or within treatments) but differed between sexes (or between treatments) (models 2 or 3 in Table 1).

**TABLE 1:** A series of multivariate mixed‐effects models to estimate sex and insecticide effects on mean‐standardised among‐individual trait variances (V).

Model	Variance structures	Description
1	[*V*_FC_ = *V*_FT_ = *V*_MC_ = *V*_MT_]	Null model—All mean‐standardised among‐individual variances were constrained to be equal.
2	[*V*_FC_ = *V*_FT_] ≠ [*V*_MC_ = *V*_MT_]	Sex effect – Mean‐standardised among‐individual variances were constrained to be equal within a sex but differed between sexes.
3	[*V*_FC_ = *V*_MC_] ≠ [*V*_FT_ = *V*_MT_]	Treatment effect – Mean‐standardised among‐individual variances were constrained to be equal within the treatment but differed between treatments.

Abbreviations: FC, females not exposed to clothianidin; FT, females exposed to clothianidin during the nymph stage; MC, males not exposed to clothianidin; MT, males exposed to clothianidin during the nymph stage.

Lastly, to estimate how early exposure to insecticides affected individual‐level viability selection on traits (body mass and activity) for each sex, we used sex‐specific multivariate mixed‐effects models (four response variables: control‐trait, insecticide‐trait, control‐lifespan, insecticide‐lifespan). In the model, no fixed effects were fitted, but individual identity was included as a random effect. In the among‐individual variance–covariance matrix (I matrix), within‐treatment among‐individual variances and covariances were estimated, while cross‐treatment among‐individual covariances were set to zero since individuals were not able to experience both treatments. We then calculated among‐individual correlations between the focal traits and survival; these correlations serve as proxies for the viability selection acting on the labile traits under laboratory conditions (Dingemanse et al. 2021).

## 3 Results

**FIGURE 1:**
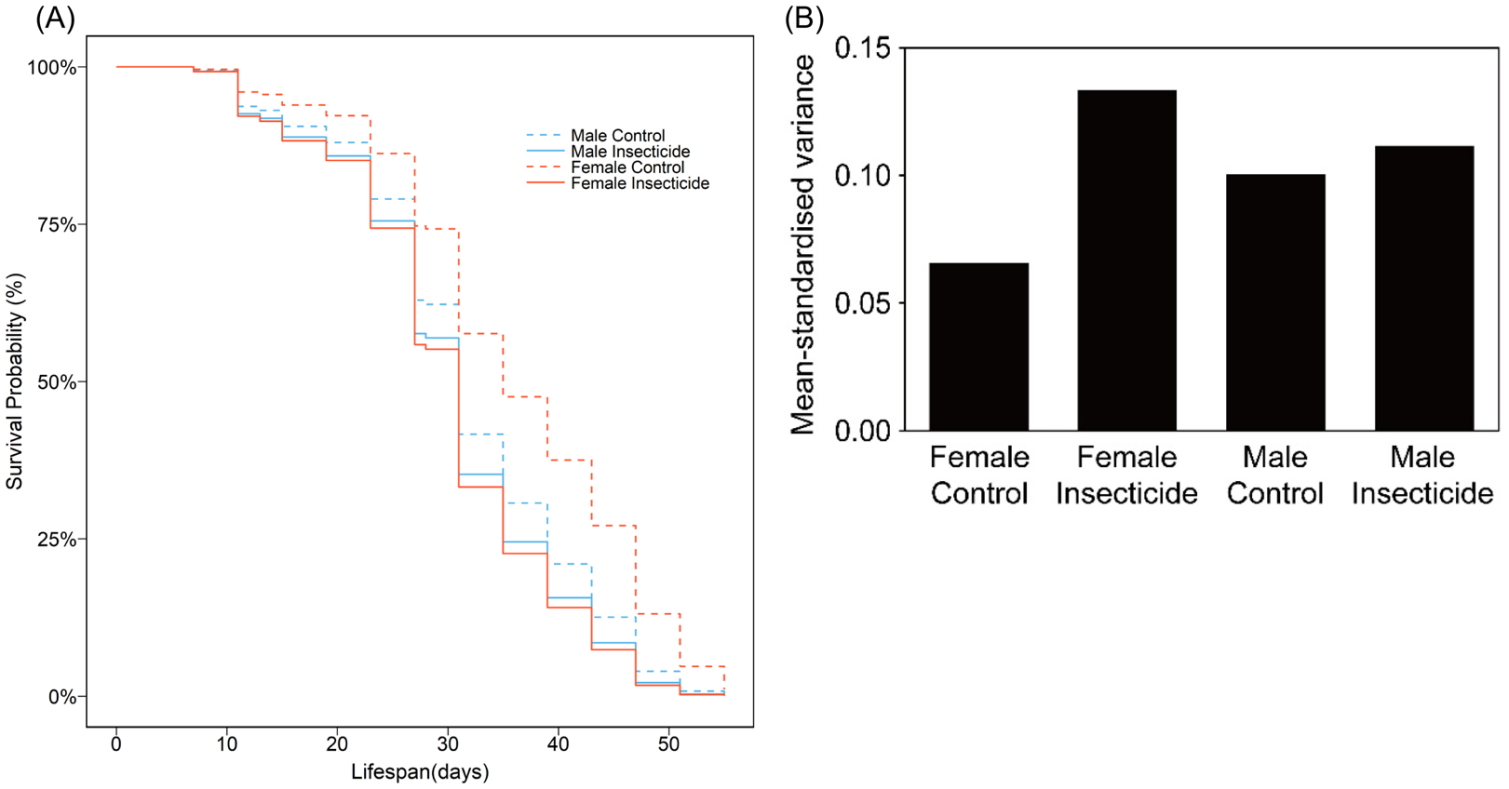
(A) Survival of adult *Gerris latiabdominis* males and females exposed to clothianidin (insecticide group) or not exposed (control group) during the nymph stage. (B) Mean‐standardised among‐individual variances in lifespan across four combinations of sex (male vs. female) and treatment (control vs. insecticide).

Females survived significantly longer than males (hazard ratio (SE) = 1.63 (0.23), *z* = 2.09, *p* = 0.04; Figure 1A). Individuals exposed to clothianidin during the final nymphal stage (insecticide group) faced approximately double the instantaneous risk of death compared to those that were not exposed (control group) (hazard ratio (SE) = 2.07 (0.25), *z* = 2.95, *p* = 0.003; Figure 1A). However, the increase in mortality risk associated with clothianidin exposure did not differ significantly between sexes (hazard ratio (SE) = 0.58 (0.34), *z* = −1.61, *p* = 0.11).

Clothianidin exposure during the final nymphal stage significantly increased the mean‐standardised variance in lifespan (Estimate (95% CI) = 0.36 (0.04, 0.67); Figure 1B). Although this increase in variance after clothianidin exposure tended to be more pronounced in females (+203% increasing from 0.07 to 0.13) compared to males (+111% increasing from 0.10 to 0.11), the interaction between sex and treatment regarding variance changes was not statistically significant (Estimate (95% CI) = −0.30 (−0.75, 0.16); Figure 1B).

Females were significantly heavier than males, but did not differ in activity levels (Table 2A,C). Exposure to clothianidin during the final nymphal stage resulted in reduced body mass for both adult males and females compared to controls (insecticide group—female: 19.0 mg, male: 13.3 mg; control group—female: 20.7 mg, male: 14.5 mg, Table 2A). However, exposure during the final nymphal stage to clothianidin did not affect adult activity levels in either sex (Table 2C). In addition, activity levels declined over repeated measures, whereas the body mass remained constant throughout (Table 2).

**TABLE 2:** Linear mixed‐effects model of body mass and activity as a function of treatment (control vs. insecticide), sex and their interaction. Exposure to sublethal clothianidin significantly reduced body mass but did not affect activity, and these effects did not differ between sexes. The parameters are provided with standard errors in parentheses.

Fixed effects	Body mass^c^			Activity^c^		
	*β* (SE)	F_NUMdf,DENdf_	*p*	*β* (SE)	F_NUMdf,DENdf_	*p*
Intercept	0.51 (0.08)	0.43_1,7.6_	0.63	0.25 (0.16)	0.01_1,45.1_	0.03
Treatment^a^	0.51 (0.10)	42.96_1,151.7_	< 0.001	0.11 (0.16)	2.54_1,150.1_	0.11
Sex^b^	−1.56 (0.01)	582.30_1,150.9_	< 0.001	−0.16 (0.17)	0.46_1,148.2_	0.50
Treatment × Sex	−0.14 (0.14)	1.05_1,151.4_	0.31	0.15 (0.23)	0.43_1,149.0_	0.51
Testing order	0.51 (0.08)	0.05_1,451.4_	0.82	−0.14 (0.05)	7.28_1,301.5_	0.007

Random effects	*σ* ^2^ (SE)	*χ* ^2^_df_	*p*	*σ* ^2^ (SE)	*χ* ^2^_df_	*p*
ID	0.15 (0.02)	148.62_0/1_	< 0.001	0.21 (0.06)	17.82_0/1_	< 0.001
Block	0.00 (0.01)	0.00_0/1_	0.50	0.00 (0.01)	0.00_0/1_	0.50
Residual	0.14 (0.01)			0.78 (0.06)		

^a^ The reference category was the insecticide treatment.

^b^ The reference category was female.

^c^ Traits were square root transformed and z‐transformed.

**FIGURE 2:**
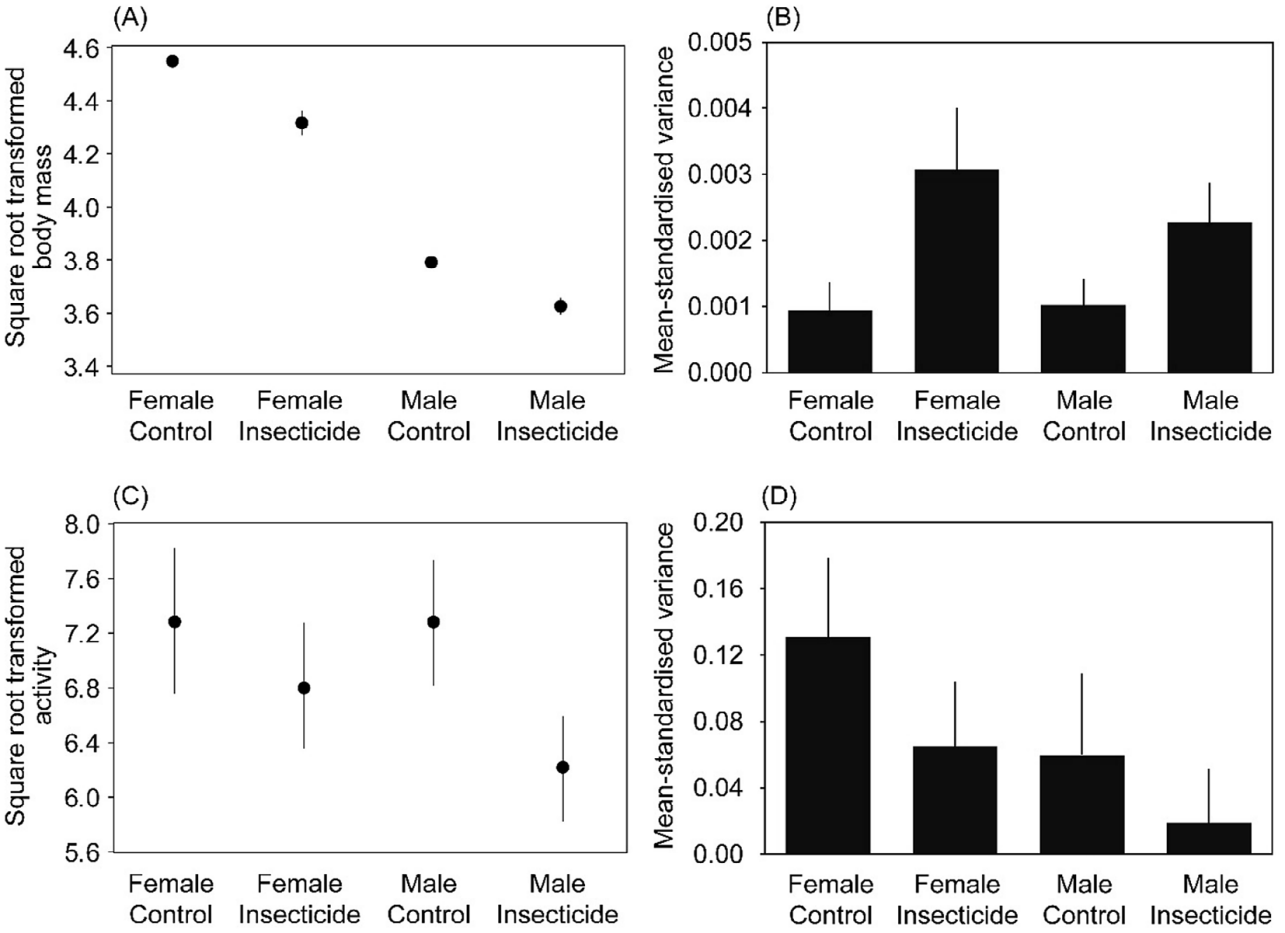
(A) Mean values of square root transformed body mass, (B) mean‐standardised among‐individual variances in body mass, (C) mean values of square root transformed activity and (D) mean‐standardised among‐individual variances in activity across four combinations of sex (male vs. female) and treatment (control vs. insecticide).

Individuals varied in body mass in both sexes across both the insecticide and control groups (control‐female: *χ* ^2^_0/1_ = 36.90, *p* < 0.001; insecticide‐female: *χ* ^2^_0/1_ = 30.06, *p* < 0.001; control‐male: *χ* ^2^_0/1_ = 50.20, *p* < 0.001; insecticide‐male: *χ* ^2^_0/1_ = 47.04, *p* < 0.001). The extent of mean‐standardised among‐individual variances in body mass did not differ between sexes (*χ* ^2^_1_ = 0.00, *p* = 0.50) but was significantly greater in the insecticide group than in the control group for both sexes (Figure 2B, *χ* ^2^_1_ = 10.64, *p* = 0.001). The increase in mean‐standardised among‐individual variances in body mass after clothianidin exposure tended to be more pronounced in females (+326% increasing from 9.42 × 10^−4^ to 3.07 × 10^−3^) compared to males (+222% increasing from 1.02 × 10^−3^ to 2.27 × 10^−3^).

Females varied in activity among individuals in both the control and insecticide groups (control‐female: *χ* ^2^_0/1_ = 10.35, *p* < 0.001; insecticide‐female: *χ* ^2^_0/1_ = 3.99, *p* = 0.02), whereas males did not show significant among‐individual variance in either group (control‐male: *χ* ^2^_0/1_ = 2.62, *p* = 0.053; insecticide‐male: *χ* ^2^_0/1_ = 0.19, *p* = 0.33). In contrast to body mass, exposure to clothianidin during the final nymphal stage did not affect the extent of mean‐standardised among‐individual variances in activity for either sex (Figure 2D, *χ* ^2^_1_ = 1.39, *p* = 0.24).

**FIGURE 3:**
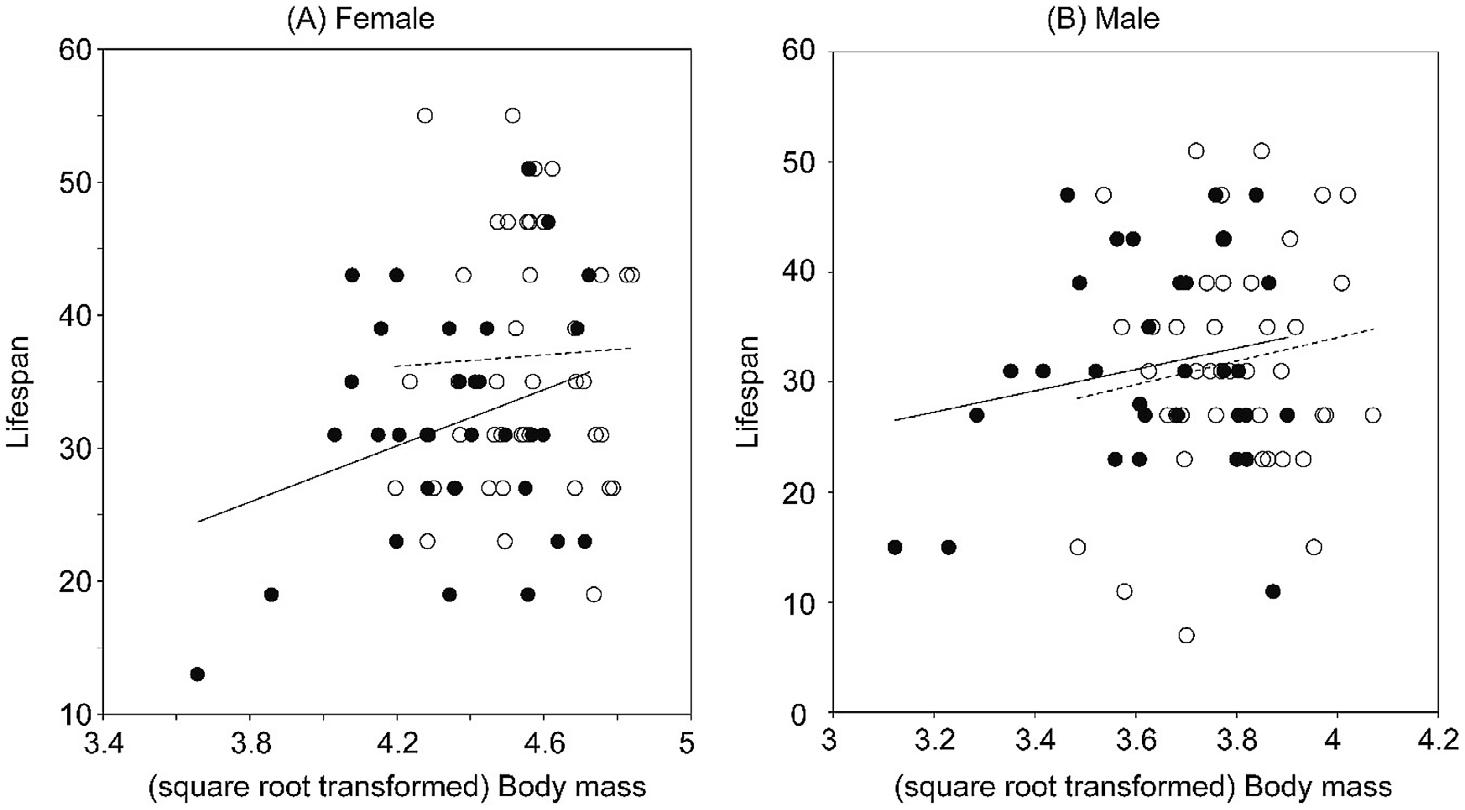
Among‐individual correlations between body mass and lifespan in the control (no clothianidin; open circles and dotted lines) and insecticide (clothianidin concentration: 7 μg/L; closed circles and solid lines) groups for (A) females and (B) males.

In females, the among‐individual correlation between body mass and lifespan was significantly positive in the insecticide group (*R* = 0.50, SE = 0.14, *χ* ^2^ = 7.16, df = 1, *p* = 0.007; Figure 3), while no significant correlation was detected in the control group (*R* = 0.05, SE = 0.19, *χ* ^2^ = 0.04, df = 1, *p* = 0.84; Figure 3). The difference in these correlations between the two groups was marginally significant (*χ* ^2^ = 3.16, df = 1, *p* = 0.075). In males, the among‐individual correlation between body mass and lifespan did not differ from zero in either the insecticide or control group (control: *R* = 0.14, SE = 0.17, *χ* ^2^ = 0.56, df = 1, *p* = 0.45; insecticide: *R* = 0.25, SE = 0.18, *χ* ^2^ = 1.60, df = 1, *p* = 0.21). Similarly, in females, the among‐individual correlation between activity and lifespan did not differ from zero in both groups (control: *R* = 0.13, SE = 0.20, *χ* ^2^ = 0.34, df = 1, *p* = 0.56; insecticide: *R* = 0.26, SE = 0.28, *χ* ^2^ = 0.56, df = 1, *p* = 0.45). In males, the among‐individual correlation between activity and lifespan could not be calculated due to the absence of among‐individual variance in male activity.

## 4 Discussion

We demonstrated that sublethal clothianidin exposure during the final nymphal stage reduced lifespan and body mass, while simultaneously increasing among‐individual variance in these traits. Although this increase in variance appeared more pronounced in females than in males, the sex difference was not statistically significant. Furthermore, positive viability selection on body mass was observed only in exposed females, although the difference in selection strength between the insecticide and control groups was marginally significant in females. These findings indicate a possibility that sublethal clothianidin exposure may contribute to the evolution of increased sexual dimorphism in body mass in *G. latiabdominis* . This study underscores the importance of evaluating sex‐specific and trait‐specific responses when assessing the effects of insecticides on non‐target insects. Moreover, we emphasise that insecticides can simultaneously influence trait means, variance, and selection, highlighting the potential to alter evolutionary trajectories.

Sublethal clothianidin exposure reduced both lifespan and body mass in *G. latiabdominis* , indicating that exposure to sublethal levels of clothianidin during the final nymphal stage was stressful for adult water striders. Insects exposed to insecticides often exhibit metabolic resistance mechanisms, such as detoxification, which impose significant energetic costs and can result in body mass loss (Sokolova 2021). For example, clothianidin exposure in fifth instar larvae led to a reduced body mass in monarch butterflies ( *Danaus plexippus* ) (Wilcox et al. 2021). Adult honey bees ( *Apis mellifera* ) that ingested non‐lethal doses of clothianidin exhibited lower body mass (Yao et al. 2018). Although we did not directly measure reproductive success, the observed reductions in lifespan and body mass likely represent substantial fitness costs, particularly given the strong association between lifespan and reproductive output documented in other water strider species (Hyun and Han 2021). Thus, exposure to sublethal clothianidin levels during the final nymphal stage may entail potential fitness costs for adults in *G. latiabdominis* .

In addition, the increased among‐individual variances in lifespan and body mass following sublethal clothianidin exposure further supports the role of clothianidin as an environmental stressor. Stressful or novel environments are known to reveal cryptic genetic variance—genetic variance that remains hidden under favourable conditions—leading to increased phenotypic variance (Badyaev 2005; Paaby and Rockman 2014; Rowiński and Rogell 2017; Schlichting 2008). For example, in a study of threespine stickleback ( *Gasterosteus aculeatus* ), environments with unfamiliar salinity levels increased the genetic variance in body size (McGuigan et al. 2011). This increased variance represents a greater capacity for adaptive evolution, potentially enabling populations to shift rapidly towards new fitness peaks under stressful conditions (Zheng et al. 2019). This finding suggests that clothianidin exposure may similarly unlock hidden variance, enhancing the potential for adaptive evolution of body mass in response to sublethal insecticide stress.

However, unmeasured container‐to‐container variance in actual clothianidin concentrations might have contributed to the increased among‐individual variances in lifespan and body mass. Because we relied on a nominal concentration of 7 μg/L and did not measure the actual clothianidin concentrations during the exposure period, heterogeneous degradation or evaporation across containers could have induced this increase in variance. To mitigate this potential artefact, we dispensed a single stock solution into identical individual containers for the insecticide treatment. As all containers were maintained under the exact same environmental conditions, any abiotic degradation would have occurred uniformly across all replicates. Therefore, container‐to‐container variance in actual exposure was likely minimal, and the increased among‐individual variance observed in the insecticide treatment can be attributed to biological responses to the insecticide stress rather than environmental heterogeneity.

While sublethal clothianidin exposure during the final nymphal stage affected adult lifespan, body mass and its variance similarly in both sexes, significant positive viability selection on body mass was observed only in exposed females. This pattern may result from a positive relationship between body mass and stress resistance, where heavier individuals might be better equipped to withstand insecticide stress and live longer. Similar patterns of positive associations between body size and stress resistance have been observed in *Drosophila* (Chippindale et al. 1996; Harshman et al. 1999; Hercus and Hoffmann 1999; Telonis‐Scott et al. 2006; but see Gibbs et al. 1997; Hoffmann and Harshman 1999; Hoffmann and Parsons 1989). In our study, some females exposed to clothianidin developed very low body mass and exhibited extremely short lifespans (Figure 3). These findings may indicate that maintaining a certain body mass could be important for somatic maintenance under insecticide stress. Females with relatively low body mass tended to die earlier, suggesting that resource limitation might constrain their ability to cope with toxicity. In contrast, heavier females with greater resource acquisition may have been better able to allocate more energy to physiological processes that alleviate toxicity effects (Houston et al. 2007).

Under insecticide stress, *G. latiabdominis* female lifespan tended to be condition dependent, whereas male lifespan was not, suggesting that the sexes may adopt different life‐history strategies under environmental stress (e.g., Martinossi‐Allibert et al. 2017, 2019). Under such stress, females—typically under stronger selection for fecundity—may make their fitness more condition dependent (e.g., linked to body mass), whereas males—often under stronger sexual selection—may prioritise traits related to mating success, sometimes at the expense of somatic maintenance, regardless of their energy reserves. For example, in Queensland fruit flies ( *Bactrocera tryoni* ), increased protein intake enhances phenoloxidase activity (an immune response indicator) in both sexes, whereas increased carbohydrate intake increases immune responses only in females (Fanson et al. 2013). Similarly, in pathogen‐infected *Daphnia magna* , females increased energy expenditure to respond to infection with greater resource acquisition, while males did not (Gipson et al. 2022). These studies suggest that males may exhibit relatively fixed and minimal investment into somatic maintenance regardless of resource acquisition, which is consistent with our finding that male lifespan was not associated with body mass under insecticide exposure. However, as our results were based on a simple comparison between a control and a single sublethal clothianidin concentration, future experiments using multiple clothianidin concentrations are needed to clarify the effects of insecticides on sex‐specific, condition‐dependent lifespan.

Unlike body mass and lifespan, exposure to sublethal amounts of clothianidin did not affect the mean or among‐individual variance in activity. While sublethal clothianidin significantly reduced body mass and lifespan, indicating clear impacts on metabolism and physiology, the activity levels of water striders may remain unaffected if striding on water surfaces requires minimal energy expenditure. This trait‐specific response to stress aligns with findings from previous studies. For example, Rowiński and Rogell (2017) reported that life‐history traits closely associated with fitness exhibited increased variation under stress, whereas morphological traits less closely linked to fitness showed none of these changes. In our study, sublethal clothianidin exposure increased variation in body mass, a trait closely associated with fitness, but had minimal impact on activity, a trait potentially less directly linked to fitness. In addition, in freshwater fish (*Pimephales promelas*), body length variation increases as water temperature rises from 23.5°C to 28°C but decreases at 31°C (Salinas et al. 2019). In contrast, the variation in the critical thermal maximum (upper thermal tolerance) tends to decrease with increasing water temperature (Salinas et al. 2019). These findings highlight that the effects of environmental stress on trait variance can differ across traits.

While our study focused on exposure during the final nymphal stage—mirroring the early‐summer agricultural application— *G. latiabdominis* in agroecosystems may also face chemical stressors during earlier, potentially more vulnerable life stages. The initial application of clothianidin in agricultural fields (April–May) typically coincides with the adult breeding season and the emergence of early‐instar nymphs. Exposure during this highly sensitive early developmental window would likely have more severe consequences for ontogeny and survival. Consequently, early‐stage or continuous exposure throughout the entire developmental period could potentially impose even stronger viability selection than the effects observed in our current study. Therefore, we highly recommend that future ecotoxicological studies investigate the effects of clothianidin exposure during the early‐instar stage to comprehensively elucidate their ecological and evolutionary consequences for *G. latiabdominis* populations.

We emphasise that the effects of sublethal insecticide exposure on non‐target species can be both sex‐ and trait‐specific. These effects, acting as environmental stressors, may lead to sex‐specific trait evolution, potentially contributing to the evolution of sex differences. Furthermore, we suggest that the impact on semi‐aquatic insects should be incorporated into assessments of sublethal insecticide effects on agroecosystems. As vital predators, semi‐aquatic insects play a crucial role in maintaining community dynamics and controlling pest populations. Our findings, which demonstrate that sublethal insecticides can impose significant stress on semi‐aquatic insects, highlight the need to broaden ecotoxicological studies on insecticide effects to include semi‐aquatic insects, extending the focus beyond solely terrestrial or aquatic insects.

## Author Contributions

**Byeongho Lee:** conceptualization (equal), data curation (equal), investigation (equal), methodology (equal), visualization (equal), writing – original draft (equal), writing – review and editing (equal). **Jiman Heo:** investigation (equal), writing – original draft (equal), writing – review and editing (equal). **Chang S. Han:** conceptualization (equal), formal analysis (equal), funding acquisition (equal), project administration (equal), supervision (equal), visualization (equal), writing – original draft (equal), writing – review and editing (equal).

## Funding

This work was supported by the National Research Foundation of Korea (NRF) grants funded by the Korea government (Grants NRF‐2022R1C1C1004303, RS‐2024‐00405751, RS‐2025‐16067311, and RS‐2025‐25442355 to CSH).

## Ethics Statement

Experiments on insects do not require approval from the ethics committee of Kyung Hee University. Korea. However, our design did seek to minimise the number of individuals used and our protocols were designed to minimise disturbance of the animals.

## Conflicts of Interest

The authors declare no conflicts of interest.

## Supporting information


**Table S1:** Mortality of *G. latiabdominis* nymphs after 1 week of exposure to insecticide solutions with varying concentrations of clothianidin.
**Table S2:** Mortality and paralysis responses of *G. latiabdominis* after 1 week of exposure to insecticide solutions or pure clothianidin with varying concentrations of clothianidin.
**Text S1:** LC50 test of insecticides, including 8% clothianidin.
**Text S2:** Comparison of the effects of pure clothianidin and commercial insecticide formulations (including 8% clothianidin and surfactant) on mortality and paralysis response in *G. latiabdominis* .
**Text S3:** Statistical analyses.

## Data Availability

Data and statistical codes are available at https://doi.org/10.6084/m9.figshare.31119469.
