# Supplementary material for: Sublethal Clothianidin Exposure Increases Trait Variation and Drives Sex‐Specific Selection in the Water Strider Gerris latiabdominis
Source: Ecol Evol. 2026 Aug 23;16(8):e74223. doi: 10.1002/ece3.74223 (PMC13500452; doi:10.1002/ece3.74223)
Supplement: Supplementary file 1 [file ECE3-16-e74223-s001.docx]

**Supplementary materials**

**Text S1. LC50 test of insecticides, including 8% clothianidin**

The median lethal concentration (LC50) is a widely used indicator to assess the toxic effects of insecticides on insects (Desneux et al., 2007). In our study, we conducted an LC50 test by randomly dividing 60 fifth-instar *G. latiabdominis* nymphs into 6 groups of 10 individuals each. Each individual was placed in a separate rearing container (diameter: 120 mm; height: 80 mm).

We exposed each individual to 100ml of insecticide solutions containing clothianidin at concentrations of 0 (control), 1, 5, 10, 15, and 20 μg/L. The control group was reared in 100 mL of tap water. For the insecticide treatments, we prepared a stock solution of 100 μg/L using tap water and a commercially available insecticide (containing 8% clothianidin and surfactant). This stock solution was diluted to the required concentrations immediately before use.

After one week of exposure, we recorded survival and analysed mortality data using probit analysis with the BioRssay package in R (Karunarathne et al., 2022). The mortality data (Table S1) revealed that the LC50 of the insecticide for *G. latiabdominis* was 35 μg/L.

**Table S1. Mortality of *G. latiabdominis* nymphs after one week of exposure to insecticide solutions with varying concentrations of clothianidin.**

| **Clothianidin concentration (μg/L)** | **Number died** | **Number survived** |
| --- | --- | --- |
| **0** | **1** | **9** |
| **1** | **2** | **8** |
| **5** | **2** | **8** |
| **10** | **3** | **7** |
| **15** | **5** | **5** |
| **20** | **5** | **5** |

**Text S2. Comparison of the effects of pure clothianidin and commercial insecticide formulations (including 8% clothianidin and surfactant) on mortality and paralysis response in *G. latiabdominis***

The commercial insecticide formulation used in our study contains surfactants, which may have inherent toxicity and potentially enhance the overall toxicity of the insecticide, thereby reducing the fitness of non-target insects (Mullin et al., 2016). To assess whether the observed effects were primarily due to clothianidin, we compared the mortality and paralysis responses in *G. latiabdominis* to pure clothianidin and the commercial insecticide formulation (8% clothianidin with surfactant).

We randomly divided 160 *G. latiabdominis* individuals into two groups: one exposed to pure clothianidin and one exposed to the commercial formulation. Each group was further divided into subgroups of 10 individuals. Subgroups were exposed to solutions of pure clothianidin or the commercial insecticide at concentrations of 0, 1, 2.5, 5, 10, and 25 μg/L for one week. This broad range of concentrations, extending up to 25 μg/L (approaching the LC50 of ~35 μg/L), was deliberately chosen to assess whether the surfactant enhanced toxicity at higher stress levels.

Following the exposure period, survival and paralysis responses were assessed. Paralysis was defined as individuals being unable to move, characterised by the extension of middle and hind legs stretched backwards. The results indicated that mortality and paralysis rates were similar between pure clothianidin and the commercial formulation at equivalent clothianidin concentrations (Table S2), suggesting that the observed effects were attributable primarily to clothianidin rather than the surfactant.

**Table S2. Mortality and paralysis responses of *G. latiabdominis* after one week of exposure to insecticide solutions or pure clothianidin with varying concentrations of clothianidin.**

| **Treatment** | **Clothianidin concentration(µg/L)** | **Total** | **Number died** | **Number paralysed** |
| --- | --- | --- | --- | --- |
| **Insecticide** | **0** | **10** | **0** | **0** |
| **Insecticide** | **1** | **10** | **0** | **0** |
| **Insecticide** | **2.5** | **10** | **0** | **0** |
| **Insecticide** | **5** | **10** | **0** | **0** |
| **Insecticide** | **10** | **10** | **0** | **2** |
| **Insecticide** | **25** | **10** | **2** | **0** |
| **Pure clothianidin** | **0** | **10** | **0** | **0** |
| **Pure clothianidin** | **1** | **10** | **0** | **0** |
| **Pure clothianidin** | **2.5** | **10** | **0** | **0** |
| **Pure clothianidin** | **5** | **10** | **0** | **0** |
| **Pure clothianidin** | **10** | **10** | **0** | **1** |
| **Pure clothianidin** | **25** | **10** | **1** | **1** |

**Text S3. Statistical analyses**

*Insecticide effects on lifespan*	The survival data was analysed with survival models for interval-censored observations as individual survival was monitored every four days. We used a semi-parametric Cox proportional-hazards model (using the R package *icenReg*; (Anderson-Bergman, 2017)) to assess the effect of insecticide exposure on the lifespan of male and female *G. latiabdominis*. The survival model included treatment, sex, and their interaction as independent variables.

*Insecticide effects on mean-level trait*	We used univariate mixed-effects models to test how insecticide exposure during nymph stage affected traits (body mass and activity) and their sex differences. In the model, the focal traits was fitted as the response variable. All the traits were square root transformed (which resulted in normally distributed residuals) and z-transformed (mean=0, standard deviation=1) prior to analyses. The model included testing order (covariate), sex (two-level factor: male versus female), treatment (two-level factor: control versus insecticide) and the interaction between sex and treatment as fixed effects. Testing order was mean-centred at the population level. We also fitted individual identity as a random effect.

*Insecticide effects on phenotypic variance in lifespan*	We used a double-hierarchical generalised linear models (DHGLMs; Cleasby et al. 2015) to examine the effect of sex and insecticide treatment on the extent of phenotypic variance in lifespan. DHGLMs provide a framework for simultaneously estimating fixed and random effects on both the mean (mean component) and the residual variance (dispersion component) of a trait. This allowed us to directly quantify the influence of fixed effects on phenotypic variance using single-measure survival data (Lee & Nelder, 2006). We fitted DHGLMs using sex- and treatment-specific mean-standardised lifespan as the response variable. Mean standardisation was performed by dividing each individual’s lifespan by the mean lifespan of its specific sex and treatment group. In the mean component of the model, we included sex (two-level factor: male vs. female), treatment (two-level factor: control vs. insecticide), and their interaction as fixed effects. Although we anticipated these effects to be negligible—given that the data were standardised by group means—they were retained to ensure the structural integrity of the model. Importantly, the same fixed effects (sex, treatment, and interaction) were included in the dispersion component to formally test for differences in phenotypic variance.

*Insecticide effects on among-individual trait variances*	We built a multivariate mixed-effects model for each trait to test the effect of sex and insecticide treatment on the extent of among-individual variance in each trait. Since changes in trait averages can affect among-individual variance, we standardised each trait by dividing its square root-transformed values by the treatment- and sex-specific mean (i.e., mean standardisation, (Houle, 1992)).

For analysis, we considered each mean-standardised trait measured for each unique combination for sex and treatment as a separate response variable in our analyses. This resulted in four variables per trait: the focal trait expressed in (1) females not exposed to clothianidin during the nymph stage (female-control), (2) females exposed to clothianidin during the nymph stage (female-insecticide), (3) males not exposed to clothianidin during the nymph stage (male-control), (4) males exposed to clothianidin during the nymph stage (male-insecticide). We then constructed a multivariate mixed-effects model for each trait, where we fitted above four variables as response variables. The models did not include fixed effects but included individual identity as a random effect. Variances were estimated for both the residual variance-covariance matrix (R matrix) and the among-individual variance-covariance matrix (I matrix), with covariances set to zero.

Using the multivariate mixed-effects model, we tested whether mean-standardised among-individual variance in the traits differed between sexes or treatments (Table 1). First, to compare mean-standardised among-individual variances between sexes, we compared a null model in which all the mean-standardised among-individual variances were constrained to be equal (model 1 where [V_FC_=V_FT_=V_MC_=V_MT_], Table 1) with a model in which mean-standardised among-individual variances were constrained to be equal within sexes but differed between sexes (model 2 where [V_FC_=V_FT_]≠[V_MC_=V_MT_], Table 1). Next, to test for differences in mean-standardised among-individual variances between treatments, we compared a null model (model 1) with a model in which mean-standardised among-individual variances were constrained to be equal within treatments but differed between control and insecticide groups (model 3 where [V_FC_= V_MC_]≠[V_FT_=V_MT_], Table 1).

*Selection analysis*	Lastly, to estimate how early exposure to insecticides affected individual-level selection on traits (body mass and activity) for each sex, we used sex-specific multivariate mixed-effects models. Analysing selection on labile traits requires partitioning trait variance into among- and within-individual components and estimating among-individual correlations between traits and fitness (Dingemanse et al., 2021). Such among-individual correlations between labile traits and fitness indicate the "true" selection acting on the traits.

Our sex-specific multivariate models included four (z-transformed) response variables: (1) trait value of males (or females) not exposed to clothianidin during the nymph stage (control-trait), (2) trait value of males (or females) exposed to clothianidin during the nymph stage (insecticide-trait), (3) lifespan of males (or females) not exposed to clothianidin during the nymph stage (control-lifespan) and (4) lifespan of males (or females) exposed to clothianidin during the nymph stage (insecticide-lifespan). No fixed effects were fitted, but individual identity was included as a random effect. In the residual variance-covariance matrix (R matrix), within-individual variances were estimated but within-individual covariances were set to zero. In the among-individual variance-covariance matrix (I matrix), within-treatment among-individual variances and covariances were estimated, while cross-treatment among-individual covariances were set to zero since individuals were not able to experience both treatments. For males, since the among-individual variance in activity was not significantly positive, we could not calculate the among-individual covariances between male activity and lifespan.

*Significance*	We assessed the significance of fixed effects using Wald F-tests, while the significance of correlations or variances attributable to random effects was determined using likelihood ratio tests (LRTs). The test statistic (χ^2^_nDF_) associated with the LRT was calculated as twice the difference in log likelihood between models with vs. without a variance (or correlation) with the number of degrees of freedom (nDF). When testing a variance, we assumed the test statistic to be distributed as an equal mixture of χ^2^_0_ and χ^2^_1_ (Visscher, 2006), and denoted this as χ^2^_0/1_ in our tables.

To test whether mean-standardised among-individual variances differed between sexes or treatments, LRTs were calculated as the difference in log likelihood between models 1 and 2, or models 1 and 3 (see Table 1), assuming one degree of freedom. Additionally, to evaluate differences in among-individual correlations between control and insecticide treatments, LRTs were calculated as twice the difference in log likelihood between the full model and a reduced model in which among-individual correlations were constrained to be equal between treatments, assuming 1 degree of freedom (e.g., Hyun & Han, 2021).

**References**

Anderson-Bergman, C. (2017). icenReg: regression models for interval censored data in R. *Journal of Statistical Software*, *81*, 1-23.

Cleasby, I. R., Nakagawa, S., & Schielzeth, H. (2015). Quantifying the predictability of behaviour: statistical approaches for the study of between‐individual variation in the within‐individual variance. *Methods in Ecology and Evolution*, *6*(1), 27-37.

Desneux, N., Decourtye, A., & Delpuech, J.-M. (2007). The sublethal effects of pesticides on beneficial arthropods. *Annu. Rev. Entomol.*, *52*(1), 81-106.

Dingemanse, N. J., Araya-Ajoy, Y. G., & Westneat, D. F. (2021). Most published selection gradients are underestimated: Why this is and how to fix it. *Evolution*, *75*(4), 806-818.

Houle, D. (1992). Comparing evolvability and variability of quantitative traits. *Genetics*, *130*(1), 195-204.

Hyun, H., & Han, C. S. (2021). Morph‐specific life‐history correlations in a wing‐dimorphic water strider. *Journal of Evolutionary Biology*, *34*(8), 1340-1346.

Karunarathne, P., Pocquet, N., Labbé, P., & Milesi, P. (2022). BioRssay: an R package for analyses of bioassays and probit graphs. *Parasites & Vectors*, *15*(1), 35.

Lee, Y., & Nelder, J. A. (2006). Double hierarchical generalized linear models (with discussion). *Journal of the Royal Statistical Society Series C: Applied Statistics*, *55*(2), 139-185.

Mullin, C. A., Fine, J. D., Reynolds, R. D., & Frazier, M. T. (2016). Toxicological risks of agrochemical spray adjuvants: organosilicone surfactants may not be safe. *Frontiers in public health*, *4*, 92.

Visscher, P. M. (2006). A note on the asymptotic distribution of likelihood ratio tests to test variance components. *Twin research and human genetics*, *9*(4), 490-495.
